# The Deanes’ malarias and the antimalarial drugs in 1939 and the 1940s^†^

**DOI:** 10.1590/0074-02760250232

**Published:** 2025-12-12

**Authors:** Francisco José Roma Paumgartten, Ana Cecilia Amado Xavier de Oliveira, Cláudio Tadeu Daniel-Ribeiro

**Affiliations:** 1 Fundação Oswaldo Cruz-Fiocruz, Escola Nacional de Saúde Pública, Departamento de Ciências Biológicas, Rio de Janeiro, RJ, Brasil; 2 Fundação Oswaldo Cruz-Fiocruz, Instituto Oswaldo Cruz, Laboratório de Pesquisa em Malária, Rio de Janeiro, RJ, Brasil; 3 Fundação Oswaldo Cruz-Fiocruz, Centro de Pesquisa, Diagnóstico e Treinamento em Malária, Rio de Janeiro, RJ, Brasil; 4 Ministério da Saúde, Secretaria de Vigilância em Saúde e Ambiente, Brasília, DF, Brasil

**Keywords:** malaria, tropical medicine, parasitology, antimalarial drugs, drug resistance, prophylactic treatment

## Abstract

Malaria was one of the main research themes of Leônidas de Mello Deane (1914-1993) and Maria José von Paumgartten Deane (1916-1995), a couple of parasitologists - known among their peers as “the Deanes” - who in the late 1930s and 1940s ventured into areas of the Amazon, where the disease was endemic, and of the northeastern Brazil, where an important epidemic outbreak ecloded. Despite their knowledge of the disease and the adoption of preventive measures, both contracted malaria, he the malignant tertian fever (*Plasmodium falciparum*) and she the benign form of the disease (*Plasmodium vivax*). This article describes the circumstances in which they became infected and the evolution and outcome of their malaria. The years subsequent to the period in which they became ill witnessed extraordinary progress in the pharmacological treatment of malaria. The Deanes’ two episodes of malaria are contextualised in relation to the treatment alternatives available at the time and the couple’s suspicions regarding the development of resistance to existing antimalarial drugs in view of the persistence of recurrences of the disease despite treatment.

In the dramatic and thought-provoking history of tropical medicine, one can find several examples of scientists who contracted the diseases they were studying and/or combating. This was the case of microbiologist Max Theiler (1899-1972), who contracted yellow fever while studying its agent, the flavivirus, in the laboratory.^1^ ^,^ ^2^ Fortunately, Theiler survived the disease, developed immunity to the virus, and continued his research, which led to the development of the first effective vaccine against the yellow fever in 1937. This remarkable contribution to public health earned him the Nobel Prize in Physiology or Medicine in 1951.^1^ ^,^ ^2^

A far less famous case was the accidental infection of Luiz Hildebrando Pereira da Silva (1928-2014) by *Trypanosoma cruzi* in 1961 while he was studying it at the Department of Parasitology of the Faculty of Medicine of the University of São Paulo. He reported it in his autobiographical chronicles.^3^

Although haunted for some time by the prospect of developing chronic heart disease, Luiz Hildebrando never showed late symptoms of Chagas disease.^3^

Another illustrative example is the malaria contracted by Frederick Lowe Soper (1893-1977), a leading epidemiologist at the Rockefeller Foundation, who was the director of the Cooperative Yellow Fever Service in 1938. On January 11th, 1939, when the Brazilian government established the Malaria Service of the Northeast (Serviço de Malária do Nordeste, SMNE) under a new contract with the Rockefeller Foundation (Decree-Law No. 1042/1939), Soper became for a time head of both services.

In November 1938, Soper made a brief visit to the malarial zone of Ceará and Rio Grande do Norte to assess the situation, and to prepare the stage to enforce a contract between the Rockefeller Foundation and the Brazilian government to combat the epidemic.^4^ ^,^ ^5^ Upon returning to his office in Rio de Janeiro, 12 days after leaving the malarial region, Soper felt unwell. He was struck down by a severe malaria attack. ^.^An infected *Anopheles gambiae* had probably bitten him as he passed through an area infested with the African mosquito. Later, as head of the SMNE, ironically, Soper became known for imposing iron discipline on his subordinates, including heavy penalties for those who contracted malaria by failing to adhere to a mandatory prophylactic regimen of the antimalarial drug Atebrine^®^.^4^ ^,^ ^5^

A complete list of daring scientists and sanitarians should certainly include Leônidas de Mello Deane (1914-1993) and Maria José von Paumgartten Deane (1916-1995), a couple of parasitologists known among their peers as “the Deanes”. Leônidas and Maria made significant contributions to the understanding of malaria transmission and control in Brazil. In 1939 and 1940, they studied the habits of the variety of the species *An. gambiae* that had landed and proliferated in the Northeast region spreading a lethal falciparum malaria.^6^ ^,^ ^7^ ^,^ ^8^ It must be said that In the 1960’s, it became clear that *An. gambiae* was in reality a species complex and, later, that the actual species invading the affected areas in northeastern Brazil was *An. arabiensis*.^9^ The Deanes also investigated the distribution, habits and relationships of anophelines native to the Northeast and the Amazon regions with the endemic malaria.^10^ From mid-1940s on, as head of the Section of Malaria of the Instituto Evandro Chagas, Leônidas conducted the first studies on the successful use of dichloro-diphenyl-trichloroethane (DDT) to control this endemic disease in the Amazon.^11^ ^)^ Thanks to systematic domestic spraying of DDT beginning in 1948, the annual incidence of malaria fell by more than 95% by 1952.^12^ ^,^ ^13^ In the 1960s, Leônidas ― always assisted by Maria ― began what would be one of his main lines of research for the next three decades: the epidemiology of simian malaria, its parasites, non-human primate hosts and vectors.^14^ ^,^ ^15^ ^,^ ^16^

In the late 1930s and throughout the 1940s, on successive trips to the far reaches of the country to investigate the transmission of malaria in endemic and epidemic areas, the Deanes exposed themselves to a substantial risk of contracting it, which in fact occurred under different conditions and scenarios.

This article reports how and when the Deanes were infected and the clinical evolution of their diseases. In addition, it contextualises their illnesses considering the drugs available to treat or prevent malaria in the 1930s and 1940s.

**Leônidas’s *falciparum* malaria in 1939**

As far as the authors know, the first time Leônidas publicly commented on his and Maria’s malarial infections was in a speech (“The doctor’s debt to society in Brazil”) addressed to newly graduated students from the São Paulo School of Medicine, in December 1964.^12^ On the occasion of the 50th anniversary of the Instituto Evandro Chagas, in 1986, he briefly mentioned the fact again in a chapter of the book published to commemorate the date.^17^ It was a little later, however, in a series of interviews he gave between 1987 and 1989, that Leônidas provided a full description of how this happened.^18^

In July 1938, the Major Endemic Diseases Study Service (Serviço de Estudos das Grandes Endemias - SEGE) outpost at Timbaúba was established by Evandro Chagas, who kept his assistants Gladstone Deane and Ruy Pondé there to operate it, look for autochthonous cases of kala-azar, and investigate the *An. gambiae*-transmitted malaria.^17^ ^,^ ^19^ ^,^ ^20^ The building was of rustic construction and the laboratory was simple, but it contained everything needed to diagnose cases of malaria and investigate the habits of the mosquito (Figs 1-2).^17^ Six months later, in January 1939, Evandro assigned Leônidas and Maria to join the small team working in Timbaúba. Maria had spent 1938 improving her knowledge of the clinical treatment of tropical diseases and the laboratory methods for diagnosing them at the Instituto Oswaldo Cruz hospital. On February 9th, 1939, Maria and Leônidas left Rio de Janeiro for Fortaleza and Russas, municipality of the “Vale do Jaguaribe” region, located in the “Baixo Jaguaribe” micro-region, about 162 km from Fortaleza. According to what Leônidas told interviewers, he contracted malaria shortly after arriving at the outpost.^18^

**Fig. 1:**
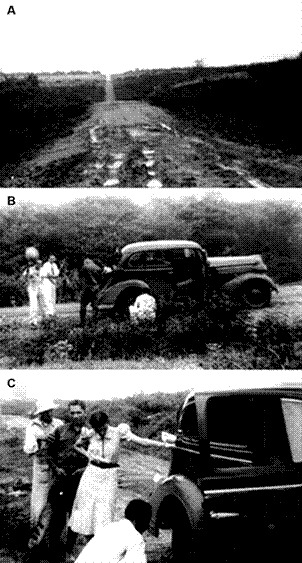
on the way to Major Endemic Diseases Study Service (SEGE) outpost in Timbaúba. (A) Road from Fortaleza to Russas in February 1939. (B) The SEGE car carrying Leonidas and Maria stopped on the road. (C) Maria looking at the flat tire change.

**Fig. 2:**
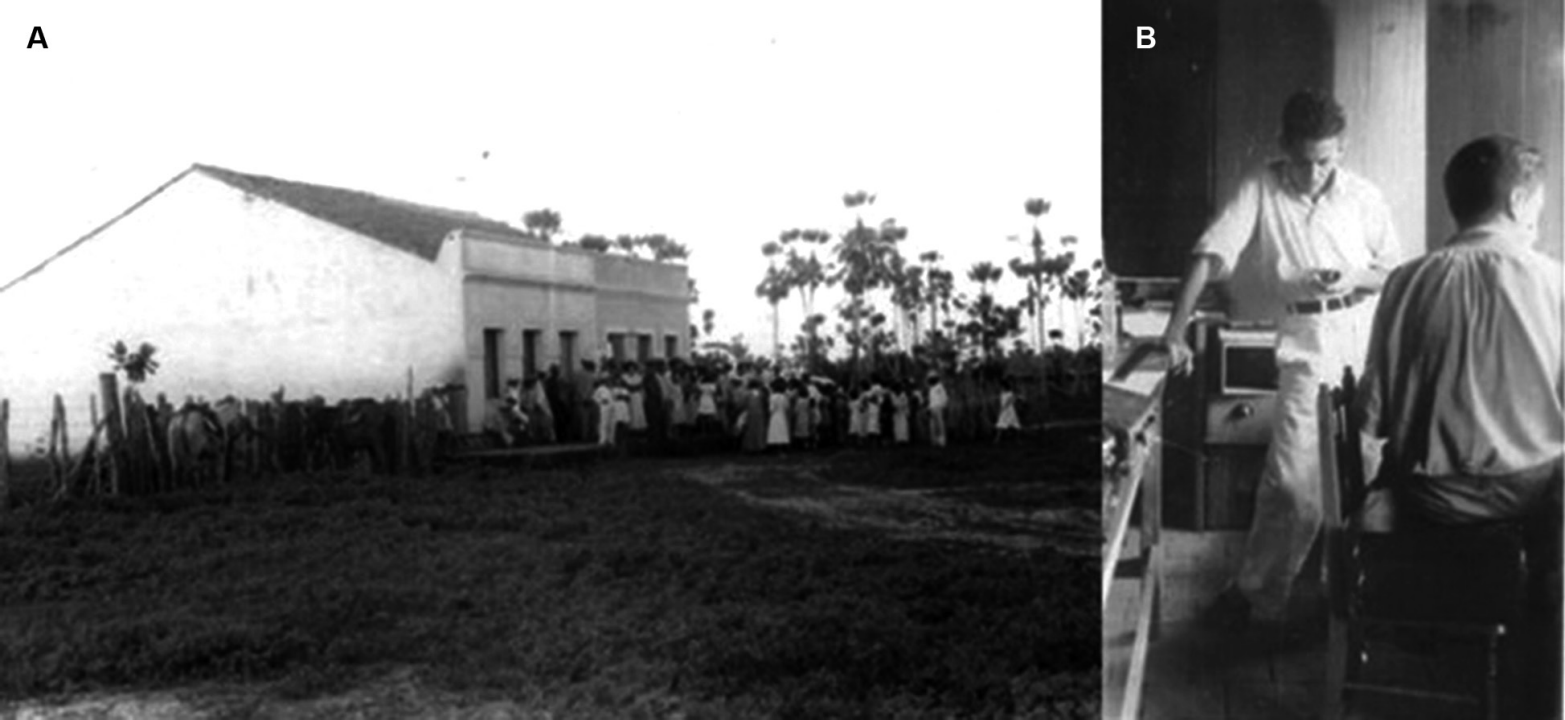
the outpost of Major Endemic Diseases Study Service (SEGE) in Timbaúba, Russas, first months of 1939. (A) Outside view of the research and ambulatory unit. (B) Leônidas (stood) and Gladstone (seated) at work in the laboratory.

*“I was examining [slides] under the microscope, and I started to feel a severe headache, seasickness and strong nausea. I told Maria that I had a terrible headache; I thought I had sinusitis, but it was an extraordinarily strong headache. Then she said ‘Oh, that’s malaria!’ I thought it could not be malaria, because there were few mosquitoes at that time. Moreover, we had just arrived; we had been in the endemic zone for 15 days. Maria took my blood and went to examine it [under the microscope]. While she was taking my blood to examine it, I lost consciousness. It was very difficult because there was no injection of Atebrine* ^*®*^ *”*.

The fulminant attack of malignant tertian fever left him unconscious for almost three days.^17^ ^,^ ^18^ When the symptoms subsided, one of the post employees carried Leônidas in his arms to a boat, which transported him to a point on the Jaguaribe River where the Russas-Fortaleza road was passable. In the capital of Ceará, Leônidas continued his antimalarial treatment and rested in bed, instead of the hammock as he usually did in the backcountry, before returning to Timbaúba (Fig. 3).

**Fig. 3:**
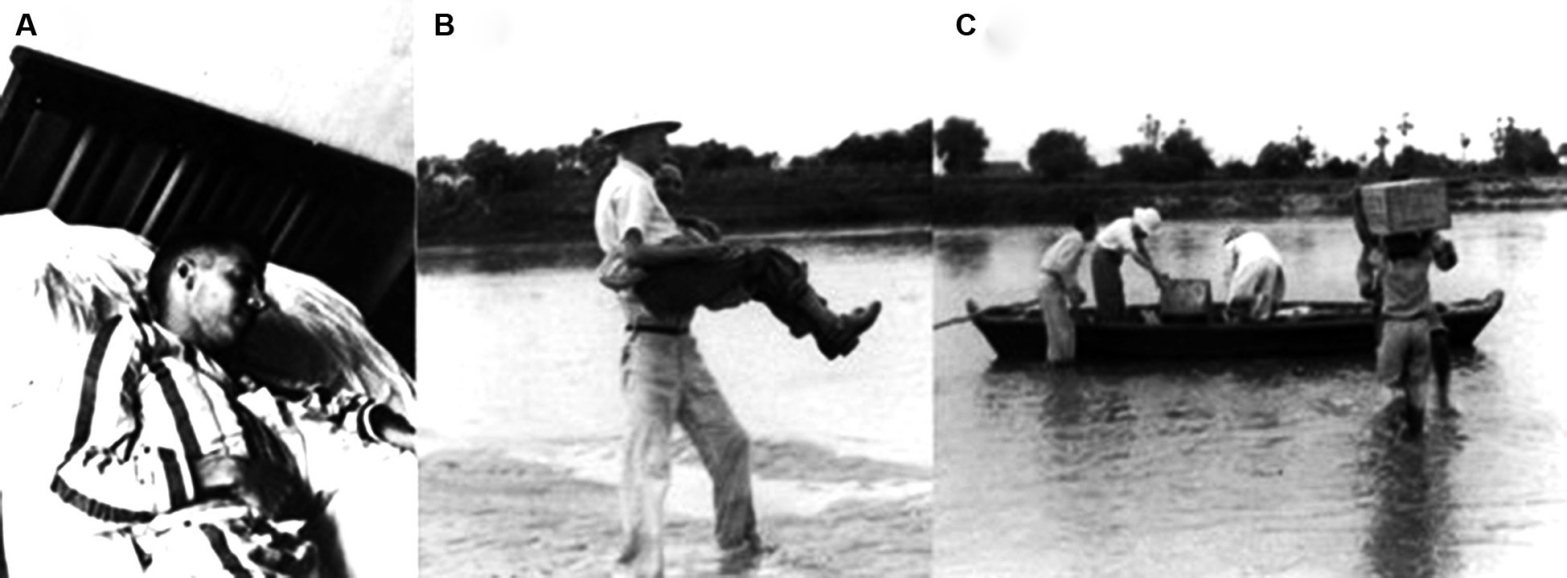
Russas, Ceará, March 1939. (A) Leônidas suffering from severe *falciparum* malaria. (B-C) On the way to Fortaleza, one of the employees of the post carries Leônidas in his arms to a boat on the Jaguaribe River.

Evandro Chagas confirmed the severity of Leônidas’ malignant tertian fever in the daily notes of relevant events ― a kind of professional “diary” ― that he kept throughout 1939.^21^ On March 13th, he commented that Leônidas “*was in very precarious conditions*”, with “*repeated cardiac collapses*” and, the following day, he made it clear how much he felt uncomfortable with his assistant.

*“[…] he set a bad example to subordinate employees by failing to carry out prophylactic treatment; then he once again carried out experiments on himself, which we have long since declared irregular and strictly prohibited […]”*.

Evandro fined Leônidas “*for not adhering to Atebrine* ^*®*^ *prophylaxis*” by deducting three days of his salary.^17^ Wladimir Lobato Paraense (1914-2012) told the episode in an interview to the Fiocruz’s Oral History Program and repeated the story in an article in memory of Leônidas.^22^ ^,^ ^23^ According to Lobato’s remembrances:^22^

*“Evandro found out and didn’t believe it, he thought he had taken Atebrine* ^®^ *and stopped on purpose to study* [the drug] *elimination and do scientific work and that the reputation of the service was at risk, I don’t know what... and he fined the guy’s salary, he fined his salary”*.

Leônidas explained with his own words the circumstances that led him to interrupt the prophylactic treatment with Atebrine^®^:^18^

*“Before going there, I was here in Rio, and I took a full dose of Atebrine* ^*®*^ *. Then, I did a test every day to see how much of the substance I had in my urine. I went to the Northeast, and 15 days later, I still had Atebrine in my urine, so I was sure that I would not catch malaria. Nevertheless, I did catch it. Although I still had some Atebrine* ^*®*^ *in my urine, it was decreasing, but I still had it.”*

Unlike SMNE, Evandro’s SEGE had not formally introduced a mandatory Atebrine^®^ prophylaxis regimen, and Evandro may have overreacted to this setback. Lobato suggested that, at that time, a sequence of small disagreements had strained the previously good relationship between the leader and his dependable assistant to the point that Evandro, in a fit of unrestrained aggressiveness would have “*called Leônidas to fight outside the research post*”.

At any rate, Lobato was not present in Russas, and thus he based his narrative on rumors he had heard at the Instituto Oswaldo Cruz in Rio.

The very severe headache, which rapidly progressed to impaired consciousness suggests that Leônidas had cerebral malaria, a life-threatening complication of *falciparum* infection.^24^ ^,^ ^25^ and, quite impressively, with a very aggressive and fast progression from the first symptoms to unconsciousness. When he fell ill and collapsed, his brother Gladstone Deane was not at Timbaúba; he had left Russas to collect the researchers’ salaries, and the research funds needed to keep the outpost running. To make a dramatic situation even worse, the Timbaúba post was isolated due to a major flood in the Jaguaribe River basin. Only Maria, Leônidas and some non-medical assistants had remained there.

Then Maria faced a challenging situation. Leônidas’s clinical condition was rapidly deteriorating, and it was not feasible to administer pills to an unconscious patient. At the time, she did not have access to injectable formulations of quinine hydrochloride or quinacrine (Atebrine^®^), which the SMNE provided in limited quantities to its own staff and to SEGE for the most serious cases, such as the one presented by Leônidas. Maria then crushed some Atebrine^®^ (quinacrine) tablets and improvised an injectable formulation that she administered to the patient, a quick intervention that saved his life. Later, during World War II (WWII), several clinical trials demonstrated the efficacy of intramuscular quinine or quinacrine in some cases of cerebral malaria.^25^

SMNE-adopted therapeutic and prophylactic antimalarial regimens

The malignant tertian struck Leônidas, when the Deane brothers and Maria were working under Evandro’s leadership for the Instituto de Patologia Experimental do Norte (IPEN) and the SEGE, not for the SMNE, a service administered by the Rockefeller Foundation and created on January 11th, 1939.^26^ The SMNE hired Leônidas and Gladstone Deane on June 14th, and Maria two days later, on June 16th, 1939.^4^

The primary objectives of the SMNE, as reported by Soper and Wilson, were “*the prevention of the spread of gambiae and its eradication from Brazil*.” However, the morbidity and mortality from malaria within the infested area were so high that the SMNE unavoidably had to treat the population until a drastic reduction in the vector caused the decline and disappearance of the disease.^4^ The SMNE routinely dispensed antimalarial drugs for all cases of acute febrile illness, even before parasitological examination confirmed infection, but the service did not adopt prophylactic administration of antimalarial drugs even in heavily infested areas, except to its own personnel.^4^

The standard therapeutic regimen consisted of oral administration of quinine tablets (sulfate or hydrochloride salts) for seven days, or Atebrine^®^ tablets for five days. SMNE sometimes added Plasmochine (pamaquine) to the therapeutic regimen (*i.e.*, plasmochine plus quinine, or plasmochine plus Atebrine^®^) used in patients living on the border of infested areas because of its gametocidal activity. The daily doses of quinine and Atebrine^®^ were age-adjusted; for those patients aged 13 or more years they were 1.25 to 1.5 g/day (five to six tablets of 250 mg) and 300 mg/day (three tablets of 100 mg) for quinine and Atebrine^®^, respectively.^4^ Despite their adverse effects, particularly in the case of Atebrine^®^, owing to their effectiveness both drugs were generally well accepted for short-term treatment of patients in highly malarial areas. The high spontaneous demand for Atebrine^®^ in malarial areas and the precautions taken by SMNE are consistent with its good acceptance by the affected population.^4^

*“[Atebrine* ^*®*^ *] was seldom given out for future administration since tablets of this drug were in such demand that they had an exchange cash value in many of the smaller stores of the interior”*.

In the case of quinine, it was neither practical nor advisable to take the entire daily dose (six 250 mg tablets) at once in the presence of the drug dispenser guard, and so the tablets dispensed by the SMNE were dyed pink to distinguish them from other quinine-based pharmaceutical products available in Brazil, and each was stamped with the service initials and the word “grátis” (for free).^4^

The population generally accepted the unpleasant side effects of Atebrine^®^ in exchange for its greater benefit, the rapid disappearance of malarial fever symptoms. However, with asymptomatic individuals who received the drug for prophylactic purposes, the situation was quite different.

Developed by the Germans in the 1920s and introduced in early 1930s, Atebrine^®^ (quinacrine or mepacrine), an acridine derivative was the second synthetic antimalarial drug - the first one was plasmochine.^27^ ^,^ ^28^ Re-synthesised by the Americans, during WWII, quinacrine replaced quinine for use as an antimalarial prophylaxis and to treat soldiers infected with malaria - most notably in the South Pacific and the Mediterranean military operation theaters.

The most conspicuous and odd side effect of Atebrine^®^ was the yellowing of the skin, which could eventually be mistaken for jaundice. Leônidas himself used to narrate how embarrassed he felt, although it amused him, when the street kids made fun of him and of the technicians from the Programme to Combat Malaria or from the companies involved in the construction of the railways, calling them ‘AMARELÃO” (“Big Yellow”!) when they saw them passing in the street, as happened to him several times. Other common adverse effects of the drug were fatigue, abdominal discomfort, cramps, nausea, diarrhoea, headache, dizziness and sleeplessness. A relatively rare but serious adverse effect associated with a continuous use of Atebrine^®^ is the emergence of psychotic reactions.^29^ ^,^ ^30^

Owing to its considerable toxicity and slow elimination rate from the body, it is necessary to alternate periods of Atebrine^®^ intake with no-drug rest periods. Thus, the prophylactic scheme adopted by SMNE to protect its working force was two 100 mg tablets daily for three consecutive days followed by a six-day rest period without medication.^4^

Atebrine^®^ tablets were part of the set of essential items periodically supplied by the SMNE to its agents for conducting fieldwork. In this regard, Leônidas’ report for June 16th, 1939, recorded:^31^

*“At the service headquarters [Fortaleza] [...] we received a hammock, a mosquito net, a travel bag, a 10x lens and 30 Atebrine tablets for personal use…”*

The thirty tablets by the service were enough for forty-five continuous days of chemical protection.

Fred Soper and David Bruce Wilson (1894-1963), head and field director of SMNE, respectively, knew that the mandatory Atebrine^®^ regimen did not completely prevent infection, yet it attenuated malaria morbidity and mortality.^4^

*“The results of prophylactic treatment of [service] personnel have shown that administration of antimalarial drugs will not prevent infection where there is a lot of malaria and an efficient vector, but it will control the symptoms in the great majority of cases and will allow field personnel to keep working, which in other conditions [without prophylactic treatment] would be impossible”.*

They also knew that penalties for noncompliance were necessary to ensure adherence to chemoprophylaxis. Thus, SMNE imposed a fine equivalent to one day’s work for those who contracted malaria. In addition, days on which the employee was absent from work due to illness were deducted from his or her salary. Notwithstanding the penalties, 725 SMNE employees fell ill with malaria, 590 during the first 18 months and 135 in the following 18 months of the 1939-1942 anti-*gambiae* campaign.^4^

**Development of *Plasmodium* sp tolerance or resistance to antimalarial drugs**

As early as 1910, Arthur Neiva (1880-1943), responsible for an antimalarial campaign in a marshy area near Rio de Janeiro, observed that the parasites infecting workers subjected to a mandatory prophylactic regimen with quinine, became, over time, less sensitive to this antimalarial.^32^

*“The doses of quinine and the frequency of its administration, which were initially sufficient to combat malaria, later became insufficient, making it essential to increase them to achieve the same goal”*.

This field observation led Neiva to infer that, after continuous exposure to quinine, part of a local population of *Plasmodia* developed “resistance” to this antimalarial drug. It should be noted that, although what Neiva noticed may, somehow, have appeared to correspond to or include a host related phenomenon, the need of higher doses of drugs to treat an episode of malaria results from the emergence of parasite isolates that developed, through genetic mutations, reduced sensitivity (resistance) to the effect of the antimalarial and from the selection of these populations by the use of inadequate (insufficient) doses of the drug. Nevertheless, although the host does not become “resistant” to the drug, even after continuous or prolonged exposure to it, host factors may play an important role in the way an individual responds to the antimalarial drug: his/her degree of immunity to *Plasmodium*, acquired through previous exposures to the parasite; coinfection with other pathogens, including HIV; use of immunosuppressive drugs; and genetic polymorphisms that may explain differences in the degrees of host’s natural protection against malaria or in the way the body processes (breakdown and elimination) and responds to antimalarial drugs. Variations in the genetic background of Cytochrome P450 2D6 (CYP2D6), for example, influence the metabolism of primaquine, an 8-aminoquinoline used to prevent relapses of *vivax* malaria, affecting how this drug is metabolised and impacting treatment outcomes in a given individual.^33^

In their account of their trip to the Madeira-Mamoré region in 1943, the Deanes referred to the failures and sad long-term health consequences of a mandatory prophylactic regimen with quinine imposed to keep the workforce fully functioning during the railroad construction. So, they seemed to believe that with prolonged exposure of parasites to quinine, the preventive regimen would tend to become less effective.^34^ Toxicity related issues could also be in their worries…

*“The region is famous for being one of the most malaria-affected regions in the world. Malaria was so common and intense during the construction of the railway that workers and all other employees were required to take quinine daily, and even so, many fell ill and died. Even Oswaldo Cruz was called in to give his opinion on how to reduce the damage caused by malaria. Even after the completion of the railway, the use of quinine by the employees continued. Many took quinine daily for years without interruption, and several cases of poisoning occurred, some of which resulted in permanent deafness and psychosis, some of which were permanent”*.

In 1943, possibly with Neiva’s communication in mind, the Deanes suspected that prolonged exposure to mepacrine could also have led to the emergence of Atebrine^*®*^ -resistant strains of *P. falciparum* in some areas:^34^

*“[...] while we were there, a SESP employee presented with malignant tertian fever that high doses of Atebrine had not been able to eliminate by the time we left the city; this employee was performing preventive atebrinization, which may have created* [or selected] *a sample of plasmodium resistant to this medication, making treatment difficult. On a previous excursion, when we were in Mulata, Monte Alegre, we had the opportunity to observe the same thing, prolonged preventive atebrinization or insufficient curative treatment of the residents, creating in them a later resistance of the plasmodium to high doses of this medication”*.

The notion that unnecessary and prolonged use, or even insufficient (sub therapeutic) doses, could result in the selection of drug-resistant strains of *P. falciparum* was a remarkable insight. In 1943, the Deanes anticipated what would only be suspected and proven, clinically and experimentally, from 1945 onwards

During WWII, Medical Brigadier Neil Hamilton Fairley (1891-1966) convinced the Australian Army that casualties caused by malaria could be drastically reduced by establishing a compulsory prophylactic regimen with Atebrine^®^.^35^ ^,^ ^36^ ^,^ ^37^ Imposing strict discipline, Fairley required soldiers to take one 100 mg tablet of Atebrine^®^ per day, with unvarying regularity, while they were in a malarial area. Prophylactic treatment was to continue for four weeks after leaving the endemic region. The scheme proved effective until, during the Aitape-Wewack campaign in New Guinea, from November 1944, an epidemic of malignant tertian (*falciparum*) affected the troops, despite the suppressive use of Atebrine^*®*^ .^38^ The investigation of the outbreak was meticulous and involved experiments with the parasites isolated from a group of patients who were evacuated to the Cairns base hospital. The foregoing study showed that some *Plasmodia* (*falciparum*) strains had become resistant to Atebrine^®^. According to Anthony W. Sweeney, those were the first documented cases of drug resistance of human malaria.^38^ Since then, it has been well established that *P. falciparum* may develop resistance to the main and most widely used antimalarial drugs, with resistance to the 4-aminoquinolines ― chloroquine and hydroxychloroquine ― being a classic example.

In their travel reports through the Amazon in 1943, the Deanes mentioned another strategic purpose of the mass use of antimalarial drugs that is no longer employed: the elimination of all “gametophores” in a given region, thus avoiding infection of the local vector, which would stop the epidemic. It was believed that plasmochine was a powerful gametocide and that combinations of this 8-aminoquinoline with Atebrine^®^ or quinine would be more effective than the latter two drugs alone. Therefore, they recommended to the Special Public Health Service (SESP) that the mass use of the combination of plasmochine (pamaquine) and Atebrine^®^ be adopted in Lábrea, a highly malarial area.^38^

*“It would also be useful to administer Atebrine and plasmochine in therapeutic doses to the entire population of the city; the exclusive use of Atebrine* ^*®*^ *or quinine would allow what happened in Mulatta (Monte Alegre) during last year’s epidemic to happen: the vivax gametophores disappeared, but the falciparum ones persisted, which allowed the resurgence of malaria with several malignant cases, since the distribution of the drug decreased in intensity”*

**Maria’s recurring bouts of *vivax* malaria in the 1940s**

After the closure of the SMNE on June 30th, 1942, the Deanes were immediately hired as assistant physicians by the SESP and allocated to the headquarters of the service’s Amazon Program in Belém, Pará. On September 24th, 1942, a Thursday, Leônidas noted in the report delivered to SESP:^39^

*“[Belém] - Having fallen ill with malaria M. P. Deane, we worked on breeding anophelines...”*

This was the first record of malaria contracted by Maria. In the previous weeks, the Deanes had conducted home inspections and collected anopheline larvae and adults in the surroundings of Belém, where, a few days earlier, SESP guards had captured many *darlingi* specimens.

In September 1943, Maria contracted malaria again, this time on the Island of Marajó (Figs 4-5). This second infection occurred in an unusual way, as reported by her husband. The Deanes were aboard the SESP boat Pará, anchored in the middle of the Arari River, far from the shore, to prevent the numerous mosquitoes from disturbing the work on the deck of the vessel that served as a dormitory and laboratory for the researchers. Leônidas and the SESP guards captured the mosquitoes on land ― with portable suction catchers ― and brought them alive to the boat inside a small wire cage so that Maria could dissect them, looking for the presence of sporozoites in the salivary glands.^18^

**Fig. 4:**
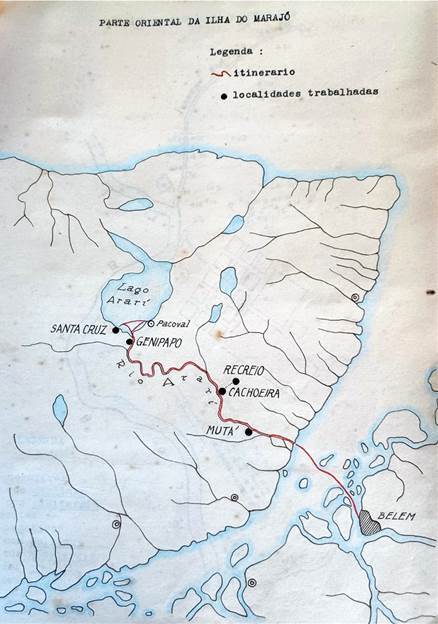
sketch drawn by Leônidas of the itinerary of the Deanes’ trip aboard the boat Pará from the Special Public Health Service (SESP) to the Island of Marajó on September 19-29, 1943.

**Fig. 5:**
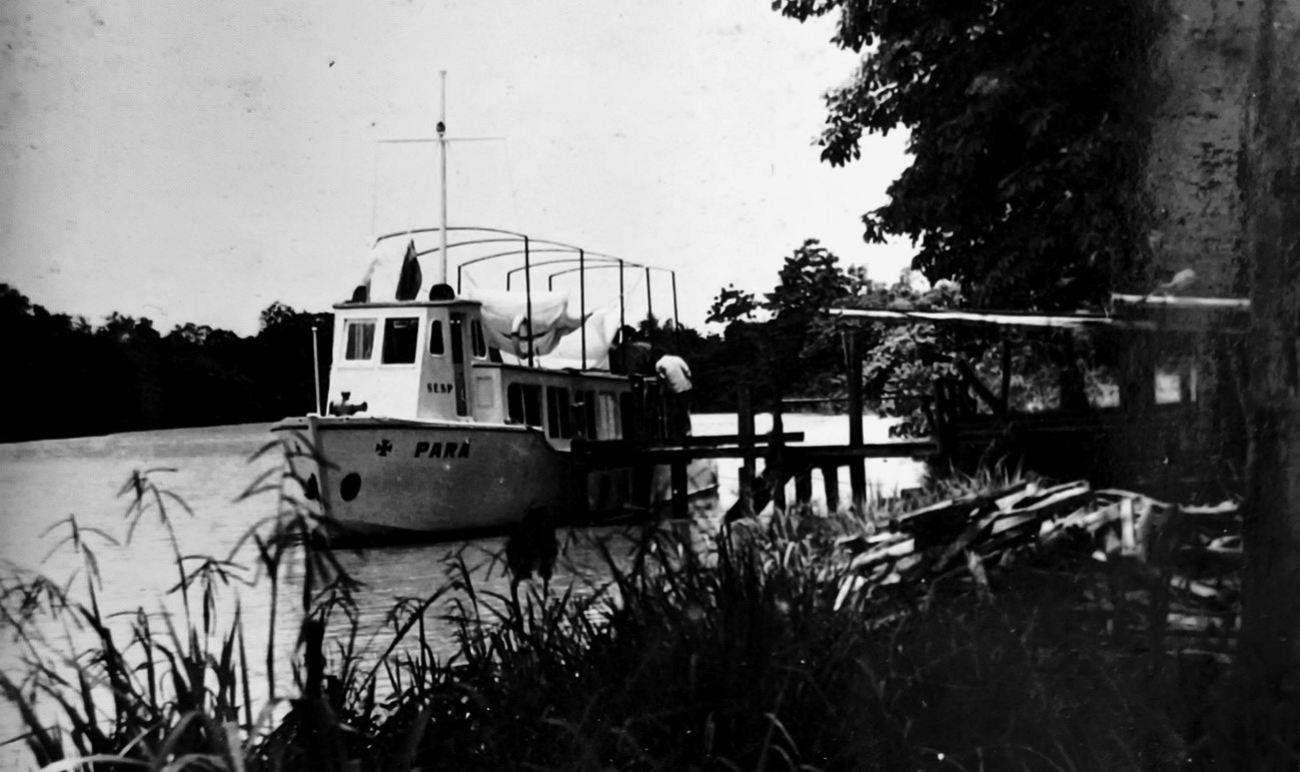
Special Public Health Service (SESP) laboratory/dormitory boat Pará where Maria, bitten by an infected *Anopheles darlingi*, contracted *vivax* malaria in the middle of the Arari River, in front of Cachoeira, Marajó Island, in September 1943.

*“She went to catch a mosquito and, when she was going to put ether on it to numb it, it got loose. She saw it flying, went to catch another mosquito and felt that she was being bitten on her knee. Then she caught the mosquito that was biting her. When she examined it, she saw that it [an A. darlingi] was much infested. Therefore, she already knew that she was going to have malaria. At that time, there was no good antimalarial and Atebrine* ^*®*^ *caused severe insomnia; she could not sleep at all when she took the preventive medication. She had to work and afterwards, this uncontrollable insomnia was a very unpleasant feeling. She decided not to take anything and wait for malaria to appear. In fact, 14 days later she fell ill with malaria and was sick for a week, due to the bite of that mosquito. She even had the documentation, which was a slide with the mosquito’s salivary glands, which had the entire pedigree of the infection*.”

The episode turned out to be a natural experiment that confirmed that the *Anopheles darlingi* was the local transmitter of benign tertian fever. Maria documented the entire transmission sequence: she had identified the species of anopheles, had slides of the mosquito’s salivary glands with abundant sporozoites, and blood smear slides that were negative during the asymptomatic pre-patent period and with the presence of red blood cells full of *Plasmodium* trophozoites and schizonts when symptoms appeared (Fig. 6).

**Fig. 6:**
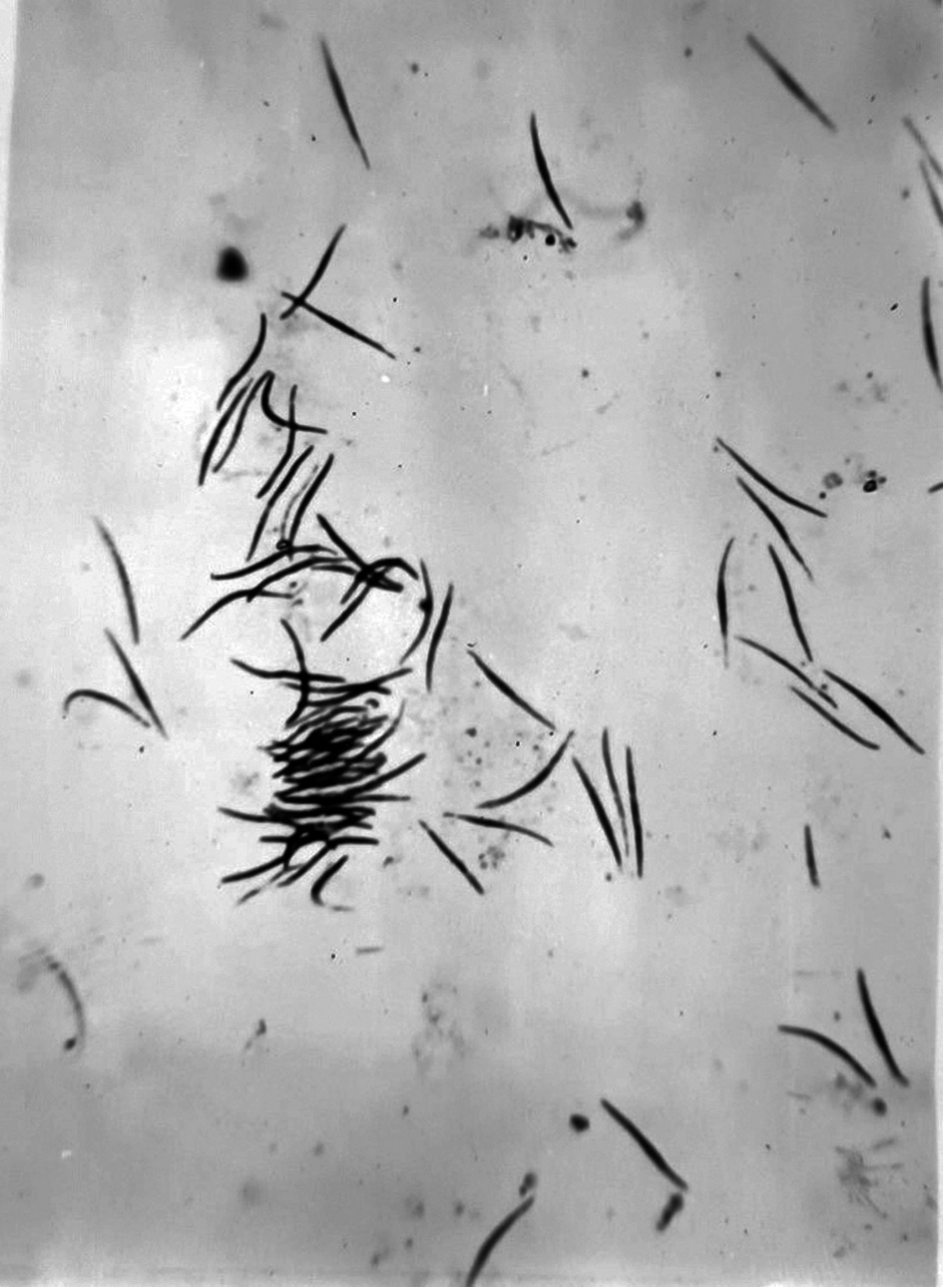
the slender and crescent-shaped sporozoites on a slide from the smear of the salivary glands of an *Anopheles darlingi* caught in Cachoeira, Marajó Island, and dissected by Maria in September 1943.

Although Leônidas stated that Maria had been ill for a week, the consequences of this unusual malarial infection continued to torment her for a long time. In the correspondence exchanged with Ottis Rembert Causey (1905-1988) in the following months, until mid-1944, there are scattered records of new attacks of malarial fever by Maria and of attempts to treat them with quinine. In principle, the new attacks might have been reinfections, recrudescence or relapses of the benign tertian fever (*vivax* malaria) contracted in Cachoeira on the Ararí River in September 1943. Based on the chronology of the symptoms and the periods of Maria’s stay in malarial regions, however, it is fair to think that these fever attacks that followed in 1944 must have been relapses of the benign tertian fever contracted on the Island of Marajó.

It may be pertinent and opportune, to a better understanding of the reader, to define the concepts of reinfections, recrudescence or relapses here: Reinfection - is a new infection in a patient that remains in the endemic area after treatment; Recrudescence - is the reoccurrence of symptoms caused by parasites remaining in the blood after an unsuccessful inadequate treatment; and Relapse - is caused by the dormant liver-stage parasites (hypnozoites) of *P. vivax* or *Plasmodium ovale*, leading to symptoms after a period without symptoms.

In a letter to Causey on January 16, 1944, Leônidas commented:^40^

*“Maria had two attacks of malaria in Fortaleza and, despite having continued taking quinine until a few days ago she had two more bouts in Valadares. Her blood was collected here and examined by the laboratory staff, who found P. vivax, which was confirmed by Dr. Amaral and by us. I am reporting this fact because the other times Maria had bouts, the blood test was always negative, which made us think it was not malaria, despite the similarity of clinical symptoms. We attributed this to the Giemsa from there, which we already thought was not good because it did not provide much contrast in color between the nucleus and plasma. The plasmodia found here in Maria’s blood clarified the fact; the slides contained abundant typical amoeboid forms, excellently stained. The Giemsa that is being used here is made in the Veiga de Carvalho laboratory, Rua Álvaro Alvim 31, Rio*.”

By the 1940s, physicians and parasitologists were generally aware of the relapse propensity of *vivax* malaria. However, at that time, they had no idea of the underlying mechanism.^37^

A major step towards clarifying this mystery was taken by the seminal work by Paraense^41^ ^,^ ^42^ and the formal demonstration by Shortt and Garnham in 1948, in non-human primates and in humans, that plasmodia had a pre-erythrocytic cycle in parenchymal cells of the liver.^43^ Three decades later, in 1976 and 1980, the final pieces of this puzzle were finally fitted together by successive works by Shute, Garnham, Krotoski and others.^44^ ^,^ ^45^ ^,^ ^46^ These studies have brought to light the long-sought explanation for relapses ― weeks or months apart ― in *P. vivax* and *P. ovale* infections. That is, there would be two populations of *vivax* (and *ovale*), one of which develops immediately in the liver and produces merozoites that invade the bloodstream after a prepatent period of one to two weeks, sometimes longer, and a second ― dormant stages or hypnozoites ― whose development remains arrested for longer periods after the sporozoite penetrates the hepatocyte.

At the time, doctors had also long known that plasmochine (pamaquine), the first synthetic antimalarial drug, introduced by the Germans as early as in 1926, tended to be more effective than quinine and Atebrine^®^ in preventing malaria relapses.^47^ ^,^ ^48^ It is unclear why Maria did not use this 8-aminoquinoline drug to get rid of recurrent malaria outbreaks. Perhaps she feared the severe adverse effects shown by some patients treated with pamaquine such as nausea, vomit, abdominal cramps and, in some patients, haemolytic anaemia. After WWII, primaquine, a more effective and less toxic 8-aminoquinoline, became available for the anti-relapse treatment of *vivax* malaria.^49^ In 1956, before the discovery of liver dormant stages of *vivax*, Alving and coworkers found that primaquine, the other antimalarial 8-aminoquinolines, and a variety of drugs, might trigger crises of haemolytic anaemia in individuals with a deficiency of glucose 6-phosphate dehydrogenase.^50^ This first study with primaquine was a milestone in the development of a new discipline, Pharmacogenetics.

In the late 1940s, Leônidas and Sutter studied in the Amazon the so-called “suppressive” treatment with a single dose of amodiaquine (Camoquin^®^), a 4-aminoquinoline compound structurally related to chloroquine (Aralen^®^). Both 4-aminoquinolines, more effective and less toxic than quinacrine, became the mainstream treatment for malaria soon after WWII. The purpose of the “suppressive” approach, or suppressive prophylaxis, is to prevent an infection from manifesting itself clinically. It differs from the causal prophylaxis that aims to prevent infection from establishing in the body and also from the antirelapse therapy (*e.g.*, to eliminate the dormant forms of the parasites in the liver).^51^ Interestingly, Leônidas and his collaborator noted that Camoquin^®^ prevented recurrences of *falciparum* malaria but not relapses of *vivax*.^51^

*“Although it is difficult to differentiate reinfections from recrudescence and relapses in endemic areas, it is believed that the cases cited constitute relapses for the following reasons: because the patients came from Belém, where endemicity is low; and because the same species was found in all attacks; and because there was only one case of falciparum with a single subsequent attack and 25 of vivax, with three, four and even five subsequent attacks. This last circumstance also shows that, with regard to relapses, camoquin acts more efficiently in falciparum infections than in vivax infections”*.

The *vivax* relapses were probably “true” relapses, *i.e*., those caused by persistent liver stages (hypnozoites) that were killed by 8-aminoquinolines but not by 4-aminoquinolines such as amodiaquine. In contrast, the *falciparum* recurrences were likely to have been reinfections or recrudescence due to failure of previous treatment.

In conclusion, the unique history of the Deanes’ malarias in 1939 and the 1940s and their aftermath disclose the limitations of curative, suppressive and antirelapse therapies with the highly toxic antimalarial drugs available at the time. The development of parasitic resistance to drugs, suspected but not proven before 1945, is also part of the story’s plot. The mandatory chemoprophylaxis adopted either with quinine or Atebrine^*®*^ then to keep the labour force working, or soldiers fighting in highly malarial areas proved to have had limited effectiveness and high costs in terms of unwanted effects and development of drug-resistant strains of *Plasmodia*.

## Data Availability

The contents underlying the research text are included in the manuscript.
