# Supplementary material for: The Deanes’ malarias and the antimalarial drugs in 1939 and the 1940s
Source: Mem Inst Oswaldo Cruz. 2025 Dec 12;120:e250232. doi: 10.1590/0074-02760250232 (PMC12711204; doi:10.1590/0074-02760250232)
Supplement: Peer review history

# Reviewer report: FIRST REVIEW ROUND - REVIEWERS COMMENTS

## REVIEWER #1

The article is original and provides relevant information on the history of malaria in Brazil, addressing the role played by two of the most important Brazilian researchers: Leonidas and Maria Deane. It is well developed, with a well-written text, a clear and informative abstract, and references and figures that are consistent with the text. The article describes the work developed by the Deanes in fighting and understanding the incidence of malaria in Northeast Brazil and the Amazon region in the 1930s and 1940s.
